# Exploring the Potential of Oral Butyrate Supplementation in Metabolic Dysfunction-Associated Steatotic Liver Disease: Subgroup Insights from an Interventional Study

**DOI:** 10.3390/ijms26125561

**Published:** 2025-06-10

**Authors:** Miloš Mitrović, Verica Stanković Popović, Sanja Erceg, Milena Perišić Mitrović, Ana Dobrosavljević, Andrej Stupar, Petra Vuković, Dušan Zlatković, Petar Svorcan

**Affiliations:** 1 Department of Gastroenterology, University Medical Center Zvezdara, 11080 Belgrade, Serbia; ana.dobrosavljevic68@gmail.com (A.D.); andrej.stupar@yahoo.com (A.S.); petravukovic94@gmail.com (P.V.); dusan.zlatkovic89@gmail.com (D.Z.); psvorcan@mts.rs (P.S.); 2 School of Medicine, Belgrade University, 11080 Belgrade, Serbia; vericasp@gmail.com (V.S.P.); milenaperisic@yahoo.com (M.P.M.); 3 Faculty of Pharmacy, Department of Medical Biochemistry, Belgrade University, 11080 Belgrade, Serbia; sanja.erceg@pharmacy.bg.ac.rs

**Keywords:** metabolic dysfunction-associated steatotic liver disease, butyrate supplementation, microbiome, inflammation

## Abstract

Metabolic dysfunction-associated steatotic liver disease (MASLD) is a common cause of chronic liver disease and is closely associated with metabolic abnormalities and cardiovascular risks. Butyrate, a short-chain fatty acid produced by gut microbiota, has the potential to enhance liver health by modulating inflammation and supporting gut barrier integrity. This study aimed to investigate and compare the effects of sodium butyrate and calcium butyrate in patients with MASLD. In this single-center, randomized clinical trial, 181 patients with MASLD were enrolled and assigned to receive either sodium butyrate (*n* = 121) or calcium butyrate (*n* = 60) supplementation at a daily dose of 1000 mg. The primary endpoint was the change in liver steatosis, measured using the Controlled Attenuation Parameter (CAP) via FibroScan^®^. Secondary endpoints included liver stiffness, biochemical parameters, hepatic steatosis and fatty liver indices, fecal calprotectin levels, stool short-chain fatty acid levels, and microbiome composition. A subgroup analysis compared responders (a ≥ 5% reduction in CAP) to non-responders. There were no significant changes in CAP values for either group (ΔCAP: sodium butyrate, 0.84; calcium butyrate, −0.23; *p* = 0.70). Sodium butyrate significantly reduced serum trimethylamine N-oxide and fatty liver index, while calcium butyrate led to a decrease in fecal calprotectin levels. Responders demonstrated a lower body mass index, higher levels of high-sensitivity C-reactive protein and HbA1c, and distinct microbiome profiles, characterized by lower abundance of *Subdoligranulum* and higher abundance of *Catenibacterium*. Although butyrate supplementation did not significantly improve liver steatosis as measured by CAP, the differing effects on metabolic and inflammatory markers suggest that there may be potential benefits for specific subgroups of patients with MASLD.

## 1. Introduction

Metabolic syndrome-associated steatotic liver disease (MASLD) is a leading cause of chronic liver disease worldwide, affecting nearly 30% of adults. Its significance is amplified by its strong association with other metabolic conditions, such as obesity, hyperlipidemia, diabetes mellitus, and cardiovascular disease [1]. MASLD encompasses a range of pathological conditions, from simple steatosis to non-alcoholic steatohepatitis, and can ultimately progress to liver fibrosis and hepatocellular carcinoma [2]. The exact pathogenesis of MASLD is not fully understood. Recent research has increasingly highlighted a connection between MASLD and disturbances in gut microbiota [3]. Researchers suggest that abnormalities in the communication system between the intestine and the liver—often referred to as the “gut-liver axis”—may play a significant role in the onset and progression of liver disease [4]. The gut and liver are interconnected through the portal venous system, meaning the liver is constantly exposed to intestinal bacteria and their byproducts, such as endotoxins and lipopolysaccharides (LPSs). The innate immune system, particularly in the liver, recognizes pathogen-associated molecular patterns primarily through Toll-like receptors [4]. Other proposed mechanisms involved in the pathophysiology of MASLD include the toxic effects of gut-synthesized ethanol, secondary bile acids, and the gut production of trimethylamine N-oxide (TMAO), all of which significantly impact hepatocyte lipid metabolism [5].

These observations suggest that supporting the integrity of the gut wall could positively influence liver health. Short-chain fatty acids (SCFAs)—mainly acetate, propionate, butyrate, and valerate—are bacterial metabolites produced through fiber fermentation [6]. Several bacterial families are primarily associated with fiber fermentation and SCFA synthesis: Bifidobacteriaceae, Prevotellaceae, Akkermansiaceae, Lachnospiraceae, and Fusobacteriaceae [7]. They may play a crucial role in maintaining the intestinal barrier by enhancing epithelial tight junctions and regulating the crosstalk between the gut and liver [8].

Furthermore, SCFA activity is not limited to the gut wall, it also has significant effects at the liver level. Most of the knowledge about the molecular mechanisms of butyrate supplementation on liver lipid metabolism comes from animal studies. Zhao et al. found that administering sodium butyrate enhances the activity of insulin-induced genes, which are powerful inhibitors of sterol regulatory element-binding protein (SREBP) transcription factors. This process also suppresses gene expression related to de novo lipogenesis [9]. Additionally, butyrate increases the phosphorylation of adenosine monophosphate-activated protein kinase (AMPK) in mice fed a high-fat diet. AMPK plays a crucial role in regulating intracellular metabolism and can directly inhibit the activity of SREBP-1, thus preventing hepatic lipogenesis [10]. Moreover, butyrate promotes the expression of the glucagon-like peptide-1 (GLP-1) receptor in the liver and inhibits histone deacetylases. This results in increased fatty acid oxidation and decreased lipid synthesis in liver cells, contributing to the reduction in hepatic steatosis [11]. Recently, studies have demonstrated that SCFAs regulate liver metabolic processes by modulating the expression of microRNAs as signaling molecules [12].

Supplementing with short-chain fatty acids (SCFAs) may be a promising therapeutic approach for treating metabolic dysfunction-associated steatotic liver disease (MASLD). However, the experience with SCFA supplementation specifically for MASLD is limited, as most studies have been conducted on animal models [6]. To date, only one recent study has investigated this treatment in humans, showing its benefits for reducing liver steatosis in this population [13]. Therefore, our study aims to expand knowledge in the field and evaluate the potential benefits and safety of oral butyrate supplementation in subjects diagnosed with MASLD. We also aimed to investigate whether there is a difference in the efficacy between sodium butyrate and calcium butyrate supplements. Additionally, we examined the clinical and laboratory factors that may predispose patients to respond to therapy, with the goal of reducing liver steatosis and inflammatory markers.

## 2. Results

**Figure 1:**
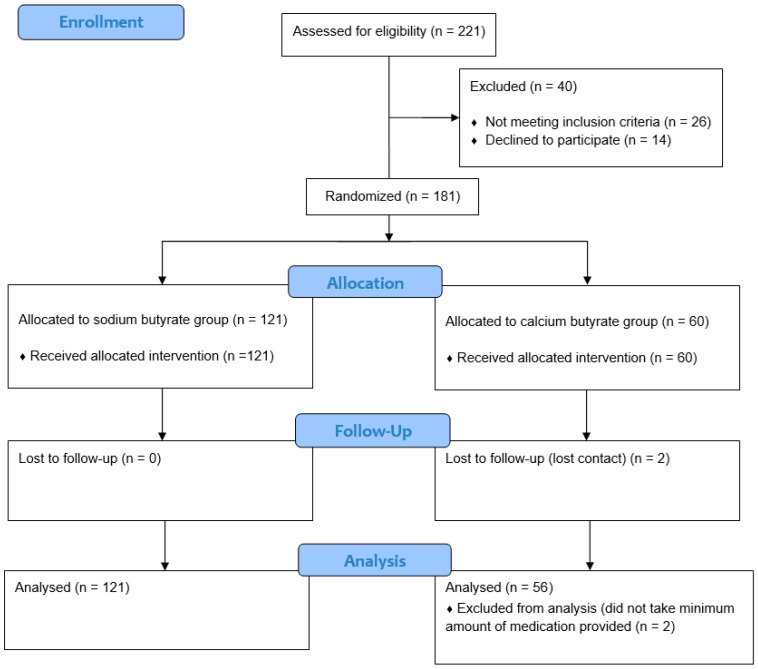
Study flow diagram.

After initially assessing 221 patients, 181 individuals completed the screening process and provided informed consent to participate in the study. Patients were randomly divided into two groups in a 2:1 ratio, favoring sodium butyrate. The first group, which received sodium butyrate, included 121 patients, while the second group, treated with calcium butyrate, consisted of 60 patients (Figure 1). This unequal allocation was driven by practical constraints related to the availability of calcium butyrate provided by the sponsor. Nevertheless, both groups met or exceeded the minimum required sample size based on power analysis, and baseline characteristics were statistically comparable (Table 1).

A total of four patients from the calcium butyrate group dropped out of the study: two did not take the minimal amount of provided tablets, and contact with another two patients from the same group was lost (Figure 1). That led to a count of 177 patients completing the study.

**Figure 2:**
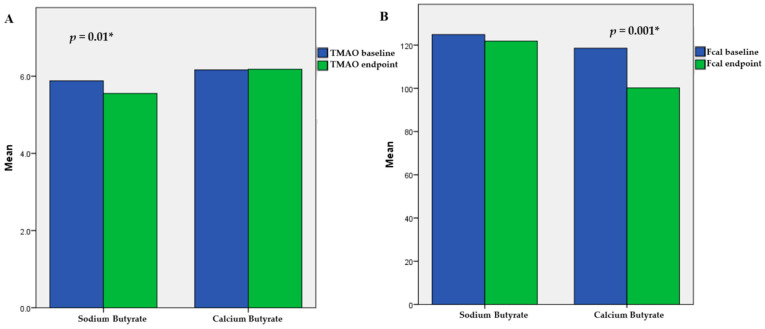
Pane (**A**)—trimethylamine N-oxide (TMAO) and pane (**B**)—fecal calprotectin (Fcal) dynamic during study period in both groups of patients. “*” statistical significance reached.

After completing the treatment protocol for 177 patients, we found that butyrate supplementation did not have a significant impact on Controlled Attenuation Parameter (CAP) dynamics in either group. For sodium butyrate, the baseline and endpoint CAP measurements were 290 dB/m and 291 dB/m, respectively (*p* = 0.81). For calcium butyrate, the measurements remained constant at 289 dB/m for both baseline and endpoint (*p* = 0.70). Furthermore, there was no notable difference in the effects of sodium butyrate compared to calcium butyrate on CAP dynamics throughout the study, with changes in CAP (ΔCAP) values of 0.84 for sodium butyrate and −0.23 for calcium butyrate (*p* = 0.70). The same could be said for liver stiffness measurements and inflammatory parameters (see Table A1). However, we observed a significant effect of sodium butyrate on trimethylamine N-oxide (TMAO) dynamics and of calcium butyrate on fecal calprotectin levels (Figure 2). No significant adverse events were observed during the study, and the tolerability of treatment was high in both the sodium butyrate and calcium butyrate patient groups (8.4 vs. 8.7, *p* = 0.74).

In addition to the CAP, LSM, and blood analysis, we assessed non-invasive measures of hepatic steatosis, including the hepatic steatosis index (HSI) and the fatty liver index (FLI) [14,15]. Overall, there were no significant changes in the HSI following the intervention (baseline: 37.7 ± 2.7; post-treatment: 37.7 ± 2.6; *p* = 0.7), nor were there notable changes among the subgroups. The FLI decreased from 74.6 ± 4.4 at baseline to 73.3 ± 13.7 after treatment, but this change was not statistically significant (*p* = 0.167). There was a modest improvement in the sodium butyrate group (ΔFLI: −4.1 ± 12.3), while changes in the calcium butyrate group were minimal (ΔFLI: +0.5 ± 15.8) (*p* = 0.036) (see Table A1). Although the HSI showed no correlation with CAP dynamics (r = 0.062, *p* = 0.413), the FLI demonstrated a strong correlation with CAP changes (r = 0.939, *p* < 0.001). This indicates a concordance between the included biochemical and clinical parameters involved and the elastographic steatosis measurements in MASLD population.

We evaluated potential changes in fibrosis risk following butyrate supplementation by calculating the Fibrosis-4 (FIB-4) score and the Non-Alcoholic Fatty Liver Disease Fibrosis Score (NFS) [16,17]. At baseline, both groups showed predominantly low fibrosis risk, with a median FIB-4 score of 1.49 for the sodium butyrate group and 1.46 for the calcium butyrate group. The median NFS at baseline was −1.13 for the sodium butyrate group and −1.21 for the calcium butyrate group (Table 1). Post-treatment analyses revealed only minor changes in fibrosis risk for both groups. Specifically, the sodium butyrate group experienced a slight increase in FIB-4 (Δ +0.02) and NFS (Δ +0.015), while the calcium butyrate group showed similar negligible increases in FIB-4 (Δ +0.023) and NFS (Δ +0.016) (see Table A1). These findings suggest that neither sodium nor calcium butyrate supplementation had a significant effect on fibrosis indices during the 12-week treatment period.

Adherence to a specific diet and physical activity regimen was high in both groups of patients and over 80% for all patients included in the analysis.

### 2.1. Mixed-Effects Model Results

To investigate whether butyrate supplementation had cumulative or interactive effects over time, we analyzed several parameters, CAP, TMAO, fecal calprotectin, HSI, and FLI, using a linear mixed-effects model. The interaction between time and treatment was not significant for CAP (*p* = 0.68), LSM (*p* = 0.55), HSI (*p* = 0.61), FIB 4 (*p* = 0.9), or NFS (*p* = 0.82), indicating that changes in these parameters were minimal and similar across treatment groups. However, we observed a significant interaction for TMAO (*p* = 0.021), FLI (*p* = 0.047), and fecal calprotectin (*p* = 0.031). This indicates that sodium butyrate resulted in a reduction in TMAO and FLI, while calcium butyrate decreased calprotectin levels to a greater extent over time. These findings support the group-specific effects noted in the simple delta comparisons (see Table A2). The mixed-effects models used random intercepts per participant and demonstrated adequate model fit (CAP dynamics model: Akaike Information Criterion—AIC = 1532.4; Bayesian Information Criterion—BIC = 1547.2). Models incorporating random slopes did not substantially improve fit and thus were not included in the final analysis.

### 2.2. Insights from Subgroup Analysis

A reduction of at least 5% in CAP was considered an adequate response to the treatment. Of the participants, 30 patients met this threshold, which included 16 from the sodium butyrate group and 14 from the calcium butyrate group. A comparison was conducted between the baseline characteristics of both the responder and non-responder groups to identify factors that could predict a positive response to butyrate treatment.

One notable difference was in body mass index (BMI), with responders having significantly lower BMI values than non-responders. The average BMI for responders was 26.1 ± 1.7, while for non-responders it was 27.8 ± 1.7 (*p* < 0.001). Additionally, a higher proportion of patients with a normal BMI (≤25 kg/m^2^) was found in the responder group, at 33.3%, compared to 13.6% in the non-responder group.

Another important distinction between the two groups was their baseline high-sensitivity C-reactive protein (hsCRP) levels, which were statistically significantly higher in the responder group. The average hsCRP level for responders was 7.7 ± 4.2 mg/L, whereas it was 4.9 ± 4.4 mg/L for non-responders (*p* = 0.006). We also observed a statistically significant difference in the starting hemoglobin A1c (HbA1c) values between responders and non-responders, with HbA1c values being higher in the responder group (6.7 ± 0.4 vs. 6.4 ± 0.5, *p* = 0.037). No significant effect of baseline cytokeratin 18 fragment (CKF18), stool SCFA, or other biochemical tests on butyrate response was observed (see Table 2).

All *p*-values were re-analyzed with Bonferroni correction to account for multiple testing. After correction (approximate adjustment factor ×10), the *p*-values for BMI, hsCRP, and HbA1c were 0.010, 0.020, and 0.370, respectively. While HbA1c did not retain significance, the key trends, BMI and hsCRP, remained directionally consistent.

**Figure A1:**
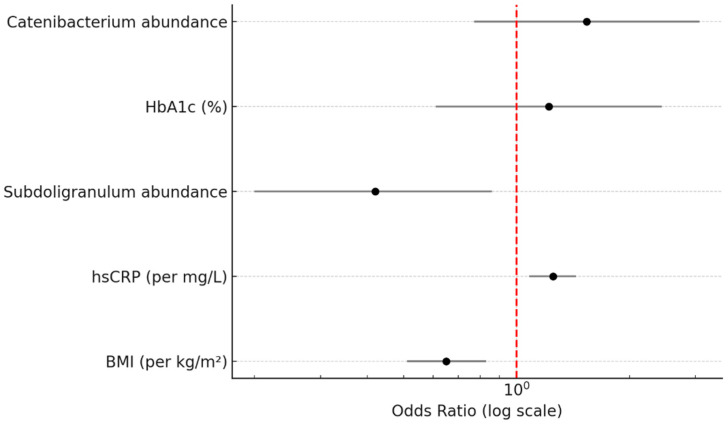
Forest plot of multivariate logistic regression analysis (HbA1c—hemoglobin A1c; hsCRP—high-sensitive CRP; BMI—body mass index).

We also conducted a multivariate logistic regression analysis to identify several independent predictors of treatment response (see Figure A1). Patients with a lower BMI had a significantly higher likelihood of achieving a ≥5% improvement in CAP, with an odds ratio (OR) of 0.65 for each unit increase in BMI (95% confidence interval [CI]: 0.51–0.83; *p* < 0.001). Additionally, those with higher baseline levels of hsCRP displayed an increased likelihood, with an OR of 1.25 for each mg/L increase (95% CI: 1.08–1.44; *p* = 0.003). However, baseline levels of HbA1c (OR: 1.22; 95% CI: 0.61–2.44; *p* = 0.57) did not retain statistical significance after adjustment in the multivariate model.

**Figure 3:**
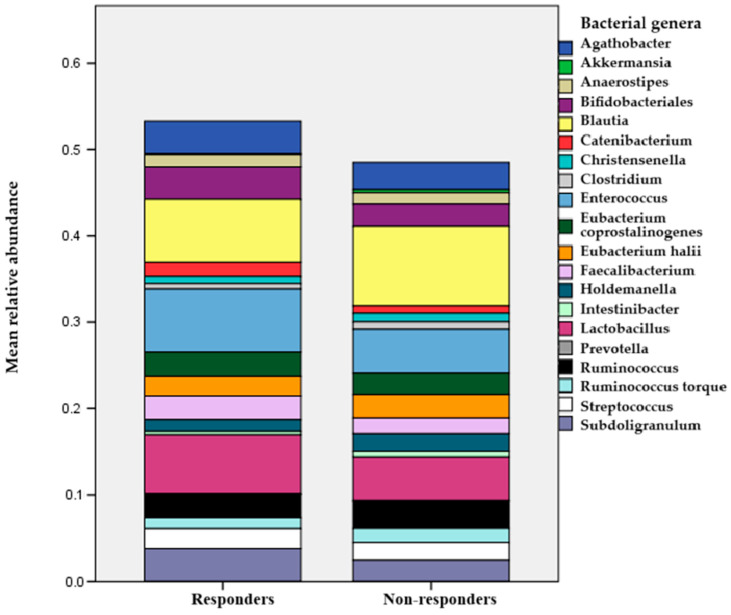
Comparison of abundance of top 20 bacterial genera in responder and non-responder stool samples.

The alpha- and beta-diversity indices of the microbiome were similar for both groups of patients. However, there were some differences in microbiome composition between responders and non-responders. After applying the Benjamini–Hochberg false discovery rate (FDR) correction, we found that the abundance of the genus *Subdoligranulum* was significantly lower in responders (*p* = 0.047). In contrast, the abundance of *Catenibacterium* was higher in responders compared to non-responders (*p* = 0.028). No other significant differences in bacterial genera and families were observed between the two groups (see Figure 3). Additionally, regression analyses confirmed that *Enterococcaceae* abundance negatively correlated with endpoint CAP values (*p* = 0.01) albeit with no statistical significance after false discovery rate (FDR) correction (Table A3).

A microbiome-focused multivariate logistic regression analysis revealed that a lower baseline abundance of *Subdoligranulum* (odds ratio [OR]: 0.42; 95% confidence interval [CI]: 0.20–0.86; *p* = 0.018) was independently associated with an increased likelihood of achieving a ≥5% improvement in the CAP score (see Figure A1). However, the abundance of *Catenibacterium* (OR: 1.54; 95% CI: 0.77–3.08; *p* = 0.22) lost statistical significance after adjustment in the multivariate model. These findings remained significant even after adjusting for clinical factors such as BMI, hsCRP, and HbA1c.

## 3. Discussion

Both sodium and calcium butyrate did not significantly affect the level of liver steatosis in the treated patient groups. However, sodium butyrate significantly reduced the level of TMAO, while calcium butyrate significantly decreased the levels of fecal calprotectin. In the group of patients who responded to butyrate treatment, lower BMI, higher hsCRP, and higher HbA1c levels were identified as positive predictors of treatment response. Additionally, a lower abundance of *Subdoligranulum* and a higher abundance of *Catenibacterium* in the stool microbiome may predispose patients to respond positively to butyrate supplementation. Overall, the tolerability of butyrate treatment was good, with no significant adverse events reported.

Despite the theoretical advantages and promising results from preclinical studies, our research found that supplementation with sodium or calcium butyrate did not significantly impact CAP dynamics or liver stiffness. The only randomized clinical trial examining the benefits of a calcium butyrate-based formula in patients with fatty liver was conducted by Fogacci et al. [13]. Our study closely resembles this trial in terms of the formulation and dosage of the butyrate formula, the similar diet followed, and the duration of the treatment period. Fogacci’s study demonstrated that butyrate supplementation has positive effects on standardized liver steatosis indices, such as the HSI and the FLI, as well as on subjective assessments of echo-sonographic liver hyperechogenicity [13]. Our study’s findings align with the Fogacci study concerning the FLI, but not the HIS [13]. The discrepancies between these indices stem from their calculation methods and the specific measures they assess. The HSI primarily relies on transaminase levels while also considering fixed parameters such as type 2 diabetes and gender. In contrast, the FLI takes into account changes in GGT, lipid levels, and incorporates clinical parameters like BMI and waist circumference. This may make the FLI more sensitive in populations with metabolic diseases, such as those in our study. Similar observations have also been reported in studies by Kim and Biciusca [18,19]. Our findings with the fatty liver index (FLI), though modest, align with recent evidence highlighting the overall limited performance of non-invasive tests (NITs) such as FLI, HSI, FIB-4, and NFS, especially for identifying significant fibrosis (MASH F2-F3). This underscores the challenge in reliably detecting subtle therapeutic changes in steatosis and fibrosis using currently available indices [20,21]

In our study, we used FibroScan^®^ 502 Touch System (Echosens, Paris, France) as the primary method for assessing liver steatosis. However, we did not find any overall statistically significant changes in liver steatosis based on CAP values, except in a specific group of patients—those with a lower BMI, higher levels of hs-CRP, or certain gut microbiome compositions. These patients may be more affected by mechanisms related to gut wall integrity or SCFA metabolism. However, several important points must be considered. First, the therapeutic effect of butyrate on hepatic steatosis may be modest and difficult to detect, particularly within a 12-week treatment period. Second, while FibroScan^®^ with CAP is a well established and validated tool for assessing liver steatosis, it may lack sensitivity in identifying subtle changes in hepatic fat content over short periods. Additionally, variations in CAP readings, which can be influenced by factors such as BMI, meal timing, and probe type, may further limit its responsiveness. Although we adjusted our statistical tests for variance imbalances, these factors should be taken into account in future trials. Incorporating more sensitive imaging techniques or adopting longitudinal study designs could enhance our ability to effectively assess changes in liver fat dynamics.

Some benefits of butyrate supplementation have been observed, particularly the positive effects of sodium butyrate on TMAO levels and calcium butyrate on fecal calprotectin levels. TMAO, which is produced by the microbiome, has gained significant attention due to its correlation with atherosclerosis and cardiovascular risk [22]. For instance, incorporating TMAO into models of traditional cardiovascular risk factors enhances the assessment of five-year all-cause mortality in individuals with stable coronary artery disease [23]. TMAO contributes to the progression of MASLD primarily by promoting the expression of SREBP-1c, a critical regulator of liver lipid metabolism that increases triglyceride synthesis and favors the progression of hepatic steatosis [24]. Lately, TMAO has emerged as a potential pharmacodynamic or response biomarker in microbiota-targeted therapies for cardiovascular and metabolic diseases [22]. However, no literature links butyrate therapy or stool SCFA levels to serum TMAO levels and MASLD development. For example, a study by *Calderon-Perez* did not identify an indirect correlation between stool SCFA levels and serum TMAO levels. Nevertheless, a more detailed microbiome analysis uncovered an inverse relationship between serum TMAO levels and the abundance of SCFA-producing bacteria, such as *Faecalibacterium prausnitzii*, in the microbiomes of hypertensive patients [25]. Based on our study, we can hypothesize that butyrate supplementation can lower TMAO levels over an extended period, which may improve cardiovascular health and slow the progression of MASLD.

Fecal calprotectin is a widely recognized marker of intestinal inflammation and is used both as a diagnostic tool and a treatment indicator, particularly for individuals with inflammatory bowel disease (IBD). During episodes of intestinal inflammation, neutrophils migrate to the intestinal mucosa and release calprotectin, a protein complex found within these cells [26]. In healthy individuals, fecal calprotectin levels typically range from 10 to 50 μg per gram of feces. However, in cases of gut inflammation, these levels can rise significantly, often exceeding 100 to 200 μg/g [26]. Such elevations can be associated with various conditions, including MASLD [27]. Pathological conditions affecting the lower parts of the digestive tract are more likely to increase fecal calprotectin levels. Currently, there is no consensus on the thresholds that differentiate normal fecal calprotectin levels from those that are considered pathological. Multiple studies have demonstrated the beneficial effects of butyrate supplementation on fecal calprotectin levels, primarily focusing on the IBD population [28]. However, only one study conducted by *Tougaard* assessed the impact of butyrate on fecal calprotectin levels in individuals with metabolic diseases, specifically type 1 diabetes mellitus. In that study, the authors found no significant benefits of sodium butyrate on fecal calprotectin levels [29]. Our findings align with the *Tougaard* study regarding sodium butyrate; however, we observed substantial positive effects of calcium butyrate. We hypothesize that these effects may be related to the slower solubility of calcium butyrate compared to sodium butyrate [30]. This slower solubility may enhance the impact of calcium butyrate in the lower parts of the digestive tract, thereby influencing fecal calprotectin levels more significantly.

In our study, we demonstrated the significant impact of butyrate supplementation on liver steatosis in patients with a near-normal or normal BMI. It is important to recognize that liver steatosis is not limited to obese individuals—19–40% of MASLD patients have a normal BMI [31]. Lean patients with MASLD share several pathophysiological mechanisms with their obese counterparts, but there are distinct features that differentiate the two groups. One hypothesis suggests that insulin resistance plays a key role in non-obese MASLD patients by increasing de novo lipogenesis and the delivery of free fatty acids to the liver [32]. Several studies have indicated that the contribution of high visceral fat and sarcopenia to MASLD is more significant than that of total body mass [33,34]. In this context, lean individuals with MASLD have relatively higher amounts of visceral adipose tissue and lower skeletal mass compared to healthy controls. Furthermore, numerous studies have reported an association between sarcopenia and complications related to MASLD, such as steatohepatitis and liver fibrosis, independent of obesity [35,36]. Finally, it is important to highlight that visceral adiposity and sarcopenia seem to act synergistically with chronic inflammation in the pathogenesis and progression of MASLD in lean patients [35]. Notably, CRP levels correlate directly with the level of visceral adiposity and negatively with appendicular muscle mass [37]. This context helps explain the observed results of our study, where the group of responders had significantly lower BMI, higher inflammatory markers, and elevated HbA1c values compared to non-responder individuals.

An important aspect of our study is the baseline analysis of the stool microbiome. We did not monitor the microbiome’s dynamics throughout the study as the intervention was not based on pre- or probiotic intervention that could modulate the microbiome composition. Our goal was to understand the baseline differences between the microbiomes of patients who responded to treatment and those who did not show significant improvement in liver steatosis. Although the microbiome characteristics were mostly similar between the two groups, we identified two key differences: the responder group exhibited a lower abundance of *Subdoligranulum* and a higher abundance of *Catenibacterium*. Many experimental studies have recognized the beneficial effects of *Subdoligranulum* on metabolic health [38]. Some of these studies suggest that its correlation with improved metabolic parameters may be coincidental or due to its tendency to thrive in healthier microbial communities and in the presence of undigested fibers [38]. However, we cannot overlook the fact that this bacterial genus is one of the primary butyrate-producing bacteria in the human microbiome [39]. These bacteria play a crucial role in gut barrier function. A study by Leclercq demonstrated an indirect correlation between the presence of *Subdoligranulum* and the influx of bacterial byproducts, especially in individuals with liver cirrhosis [40]. Furthermore, Louis established a negative correlation between *Subdoligranulum* abundance and CRP levels, as well as the Homeostasis Model Assessment of Insulin Resistance (HOMA-IR) [41]. Zhang also noted a negative correlation between *Subdoligranulum* levels and HbA1c, while showing a direct correlation with high-density lipoprotein cholesterol [42]. Additionally, we can observe the connection of Subdoligranulum to metabolic health through microbiome enrichment with this bacterium during fasting periods and the initiation of metformin therapy [43]. *Catenibacterium* is a Gram-positive, anaerobic genus belonging to the *Erysipelotrichidae* family, with one known species—*Catenibacterium mitsuokai*. This bacterium also has an ability to synthetize SCFA. There is limited literature on its metabolic functions, and the data regarding its role in disease development are mixed. Some studies suggest that it may play an important role in the elderly, providing a protective effect against frailty development [44]. Conversely, research involving patients with human immunodeficiency virus infection and chronic renal disease indicates that it could have a detrimental effect on gut barrier integrity, potentially facilitating the transfer of other bacteria and bacterial products into the bloodstream [45,46].

Our study has certain limitations, including the relatively short 12-week treatment duration, which may have limited detection of subtle hepatic improvements. Additionally, CAP sensitivity might not be sufficient for minor changes in hepatic steatosis. Future studies employing longer durations, more sensitive imaging modalities, and comprehensive longitudinal microbiome analyses are warranted.

## 4. Materials and Methods

Our single-center study was conducted as a randomized clinical trial comparing two types of butyrate supplementation: 1000 mg of sodium and 1000 mg of calcium butyrate. The study involved 170 patients diagnosed with MASLD. This diagnosis was established using a FibroScan^®^ (Echosens, Paris, France) with a Controlled Attenuation Parameter (CAP) score greater than 248 dB/m, assessed by two independent gastroenterologists (DZ, MM) [47]. Additionally, alanine aminotransferase (ALT) levels above 40 U/L for men and 35 U/L for women were required at the initial visit. Also, patients had to fulfill at least one of the cardiometabolic criteria, such as obesity, high blood pressure, or dyslipidemia. Exclusion criteria included the unwillingness to provide informed consent, alcohol consumption exceeding 30 grams per day for the six months prior to enrollment, viral hepatitis, liver cancer, bowel or liver resection, any form of bariatric intervention, previous inflammatory bowel disease (IBD), hypothyroidism, pregnancy, and breastfeeding. Patients who had taken different probiotic or antibiotic therapies within two weeks before or during the study were also excluded. The study complied with the ethical guidelines in the Declaration of Helsinki. The protocol was approved by the Institutional Ethical Committee and filed under number 1/XI/24.

### 4.1. Intervention

After signing the informed consent, all patients met with a qualified dietitian. A specific diet was implemented four weeks before treatment and continued throughout the study. The diet plan required patients to consume 20–30 kcal per kilogram of body weight, divided into 4–5 meals. It specified that 35–40% of the caloric intake should come from fats, 20% from proteins, and 40–45% from carbohydrates. Patients were provided with a cookbook containing 150 Mediterranean diet recipes. Additionally, they were asked to complete the MedQ-Sus questionnaire online or on-site to assess adherence to the planned diet [48]. All patients were encouraged to take regular daily walks, aiming for over 8000 steps. Adherence to this walking goal was monitored using the Walkmeter GPS^®^ app (Abvio Inc., San Francisco, CA, USA).

After the four-week run-in period on the specific diet, patients were randomly divided into two groups. One group received tablets containing 500 mg of sodium butyrate, which they took twice daily. The other group was given identical-looking tablets containing 500 mg of calcium butyrate, following the same dosing protocol. Computerized block randomization was performed off-site by a statistician. After randomization, the number of the therapeutic package was sent to the researchers, and the patients were issued the appropriate therapeutic package. Therapeutic packages were distributed to the patients during visits that were scheduled every four weeks on-site. The randomization data were stored on a secure server that was not accessible to patients or trial personnel until all results were finalized. Kit packaging and numbering were conducted off-site. After completing the study, patients were asked to return all unused tablets to the center. Their compliance with the therapy was evaluated by counting the returned tablets, with a maximum allowance of 20% for unused tablets.

### 4.2. Assessment

The primary objective of the study was to evaluate the effect of the treatment on CAP dynamics. To quantify liver steatosis, transient elastography with CAP determination was utilized. The examinations were conducted with the patient in a supine position, starting from the mid-axillary line and using a FibroScan^®^ 502 Touch system (Echosens, Paris, France), with the choice of probe guided by the system’s software. Each patient underwent a minimum of 10 valid examinations performed by two board-certified hepatologists (DZ and MM). The average of the two CAP values was calculated for each patient. Patients were examined at two time points: at baseline—on the day the first therapeutic package was issued—and at the endpoint—within 48 h of receiving the last therapeutic dose.

Liver steatosis was also estimated with some validated indexes, such as HSI and FLI. HSI was calculated as 8·ALT/AST ratio + BMI (+2 if woman; +2 if type 2 diabetes) [14]. FLI was calculated as [10^0.953^ × ln (triglycerides) + 0.139 × BMI + 0.718 × ln (GGT) + 0.053 × waist circumference − 15.745/(1 + 10^0.953^ × ln (trigycerides) + 0.139 × BMI + 0.718 × ln (GGT) + 0.053 × weist circumference − 15.745)] × 100 [15]. Two non-invasive fibrosis indices, FIB-4 and NFS, were calculated using baseline and post-treatment laboratory data to evaluate potential changes in fibrosis risk following treatment with sodium or calcium butyrate. The FIB-4 index was calculated according to the following formula: age (years) × AST(IU/L)/(platelet count(10 (9)/L) × √ALT(IU/L)) [16]. The NAFLD Fibrosis Score (NFS)was calculated using the following validated formula: NFS = −1.675 + 0.037 × age (year) + 0.094 × BMI (kg/m 2) +1.13 × IFG or TTDM (Yes = 1, No = 0) + 0.99 × AST (IU/L) /ALT (IU/L) − 0.013 × PLT (109/L) − 0.66 × ALB (g/dL) [17]. These indices were calculated at baseline and post-treatment, and the changes were assessed to evaluate any treatment effects on fibrosis risk.

Biochemical analyses were conducted on venous blood collected after an overnight fast. The following parameters were assessed: alanine aminotransferase (ALT), aspartate aminotransferase (AST), gamma-glutamyl transferase (GGT), total cholesterol (TC), triglycerides (TG), hemoglobin A1C (HbA1C), high-sensitivity C-reactive protein (hs-CRP), interleukin 6 (IL-6), and cytokeratin 18 fragment (CK18F).

A 2 mL serum sample from each patient was used to analyze levels of TMAO. Ultrahigh performance liquid chromatography–tandem mass spectrometry using an UPLC Acquity coupled to a MicroMassQuattro Premier XE mass spectrometer (Waters Corporation, Milford, MA, USA) was performed in order to determine the total serum levels of TMAO, according to the procedure described by Awwad et al. [49]. The samples were separated using a gradient mobile phase with a mixture of 15 mmol/L ammonium formate (pH 3.5) as solvent A and acetonitrile as solvent B. Gradient conditions were 0.0–2.0 min, 10–30% A; 2.0–3.5 min, 30% A; 3.6–4.6 min, 40% A; and 4.7–6.0 min, 10% A. We used the following settings—flow rate: 0.4 mL/min; sample injection volume: 3 μL (partial loop mode); column temperature: 30 °C; sample temperature: 4 °C; total runtime: 6 min. Blood samples for biochemical analysis and TMAO were collected at two time points: baseline and endpoint.

During the initial visit, patients were asked to provide fresh stool samples on the day the therapeutic packages were issued. Part of the stool was used for the biochemical measurement of fecal calprotectin. A portion of the stool sample was freeze-dried and subsequently used for short-chain fatty acid (SCFA) determination through capillary gas chromatography, following the method first described by Scheppach [50]. Briefly, approximately 80 mg of the dried sample was mixed with 1 ml of an extraction solution containing 0.1 mol/L oxalic acid, 40 mmol/L sodium azide, and 0.1 mmol/L caproic acid as an internal standard. SCFA concentrations were measured in the resulting supernatant using an Agilent 6890N gas chromatograph equipped with flame ionization detection and an Innowax capillary column measuring 30 m × 530 µm × 0.1 µm. Data were processed using ChemStation software (B.04.03) [51].

Another portion of the stool sample was used for microbiome analysis through 16S rRNA deep gene sequencing. Genomic deoxyribonucleic acid (DNA) was isolated from the samples using the ZR Fecal DNA MiniPrep™ kit (Zymo Research Corp., Tustin, CA, USA). After dilution to a concentration of 5 ng/µL, 10 µL samples were used for 16S rRNA amplicon sequencing, targeting the V3-V4 hypervariable region. Sequencing was performed using the NovaSeq 6000 PE250-Illumina platform (Novogene Co., Ltd., Hong Kong, China). The obtained data were filtered using Trimmomatic™ software (v0.39) and then analyzed using Quantitative Insights Into Microbial Ecology™ software (2024.7) according to the protocol described by Caporaso [52]. The composition of the microbiome was defined using Uparse software (Uparse v7.0.1001) [53,54,55]. Using R statistical software (v4.4.3), the Shannon and Simpson indices of alpha diversity were calculated, and beta diversity was estimated using a weighted UniFrac measurement.

The principal investigator consistently monitored the adverse events, clinical safety parameters, laboratory findings, and results from physical examinations. These findings were reported to the Institutional Ethics Committee. The subjective tolerability of the treatments was assessed using a Visual Analog Scale ranging from 1 to 10 [56].

### 4.3. Statistical Analysis

The minimal sample size of the intention-to-treat population was calculated using Raosoft software (4.2) [57]. The calculation was based on the known population of MASLD outpatients regularly followed-up with (*n* = 400), an expected clinically relevant effect size defined as a 5% absolute reduction in CAP from baseline, a power of 90%, an alpha error level of 5%, and an anticipated dropout rate of approximately 10%. Using these conservative assumptions, the calculated required sample size per group was approximately 60 patients. Thus, both groups met or exceeded the minimum required sample size, ensuring adequate statistical power. Also, the original sample size calculation assumed a 5% absolute reduction in CAP from baseline. Post hoc calculations revealed an observed variance in CAP changes of s^2^ = 198.4 (standard deviation, s = 14.09), translating into a Cohen’s d effect size of d = 0.076. This post hoc calculation confirmed adequate statistical power for detecting meaningful clinical differences.

A *T*-test or chi-squared test for two paired samples was conducted to identify any statistically significant differences between the baseline and endpoint CAP values, along with the biochemical parameters, TMAO, stool SCFA, and microbiome. We also analyzed the dynamics of CAP, LSM, biochemical exams, his, and FLI by calculating the absolute change between the baseline and endpoint values. To accurately account for the repeated-measures structure of the study and any potential interactions between treatment and time, we also employed a linear mixed-effects model. This model included fixed effects for time (baseline versus post-intervention), treatment group (sodium butyrate versus calcium butyrate), and the interaction between time and treatment. The linear mixed-effects model was applied to several outcomes, including CAP, LSM, TMAO, fecal calprotectin, HSI, and FLI. Linear mixed-effects modeling included a random intercept for each participant to account for individual variability. Goodness-of-fit was evaluated using AIC and BIC, with lower values indicating better model fit.

To investigate the relationship between microbiota and treatment response, we conducted non-parametric tests (Mann–Whitney U) to compare the baseline abundance of the ten most prevalent genera and families between responders and non-responders. We corrected the resulting *p*-values for multiple comparisons using the Benjamini–Hochberg FDR method. Additionally, we carried out linear regression analyses to evaluate the associations between the baseline abundance of key bacterial genera and families and the endpoint CAP values.

A decrease in CAP of at least 5% was considered a positive clinical response to treatment. A 5% reduction in CAP was selected based on previously published studies demonstrating its clinical relevance in MASLD interventions [58,59,60]. Subsequently, we compared the baseline clinical, biochemical, and microbiome characteristics, as well as stool SCFA levels, between patients who showed a positive treatment response and those who did not.

## 5. Conclusions

MASLD has emerged as one of the leading healthcare problems worldwide. There is much discussion surrounding the therapy and prevention of MASLD, with new and effective therapeutic modalities currently in the pipeline. However, the potential role of butyrate supplementation may be overlooked. Although overall liver steatosis, as measured by CAP, did not show significant improvement following butyrate supplementation, this study offers clinically valuable insights suggesting that certain patient subgroups may benefit more. Sodium butyrate significantly reduced circulating TMAO levels, which could indicate potential cardiovascular protective effects. In contrast, calcium butyrate notably decreased fecal calprotectin, suggesting a reduction in gut inflammation. Moreover, patients with lower BMI, elevated baseline inflammation (indicated by hsCRP), and specific microbiome characteristics (such as lower *Subdoligranulum* abundance) emerged as likely candidates for effective butyrate supplementation. Future therapeutic strategies should take these patient characteristics into account to enhance the clinical efficacy of butyrate therapy in managing MASLD.

## Figures and Tables

**Table 1:** Baseline characteristics of both groups of patients.

Parameter	Sodium Butyrate	Calcium Butyrate	*p*
N	121	56	-
Gender (female)	56 (46%)	28 (50%)	0.38
Age	51 ± 15	50 ± 16	0.68
BMI	27.8 ± 1.4	27.5 ± 1.8	0.19
ALT (U/L)	59 ± 10	61 ± 10	0.2
AST (U/L)	56 ± 11	57 ± 11	0.34
GGT (U/L)	70 ± 18	69 ± 19	0.9
Total cholesterol (mg/dL)	355 ± 18	351 ± 18	0.24
Triglycerides (mg/dL)	166 ± 38	167 ± 36	0.86
HbA1c (%)	6.5 ± 0.5	6.6 ± 0.5	0.09
hsCRP (mg/L)	3.2 (1.1–9.1)	4 (1.1–9.2)	0.9
Interleukin 6 (pg/mL)	42.2 ± 9.2	40.7 ± 8.5	0.31
CK18F (U/L)	248 ± 38	248 ± 32	0.93
TMAO (μmol/L)	4.3 ± 2	4 ± 2.1	0.4
Stool SCFA (mmol/L)	176 ± 36	167 ± 36	0.13
Fecal Calprotectin (μg/g)	100 (70–130)	90 (70–120)	0.23
Baseline CAP (dB/m)	290 ± 21	289 ± 19	0.82
Baseline LSM (kPA)	4.3 ± 2.4	4.9 ± 2.6	0.09
Baseline HSI	38 ± 2.6	37.1 ± 2.6	0.21
Baseline FLI	74.4 ± 4.5	75.2 ± 4.1	0.23
Baseline FIB-4	1.49 (1.10–1.87)	1.46 (1.05–1.82)	0.76
Baseline NFS	−1.13 (−1.95 to −0.32)	−1.21 (−2.02 to −0.40)	0.82

BMI—body mass index; ALT—alanine aminotransferase; AST—aspartate aminotransferase; GGT—gamma-glutamyl transferase; HbA1c—hemoglobin A1C; hsCRP—high-sensitivity C-reactive protein; CK18F—cytokeratin 18 fragment; TMAO—trimethylamine N-oxide; SCFA—short-chain fatty acid; CAP—Controlled Attenuation Parameter; LSM—liver stiffness measurement; HSI—hepatic steatosis index; FLI—fatty liver index; FIB-4—Fibrosis 4; NFS—Non-Alcoholic Fatty Liver Disease Fibrosis Score.

**Table 2:** Comparison of baseline characteristics between responder and non-responder groups.

Parameter	Responder	Non-Responder	*p*
N	30	147	-
BMI	26.1 ± 1.7	27.8 ± 1.7	* 0.001
ALT (U/L)	60 ± 13	60 ± 9	0.87
AST (U/L)	57 ± 12	57 ± 11	0.74
GGT (U/L)	68 ± 20	71 ± 18	0.583
Total cholesterol (mg/dL)	350 ± 20	354 ± 17	0.27
Triglycerides (mg/dL)	166 ± 38	167 ± 36	0.54
HbA1c (%)	6.7 ± 0.4	6.4 ± 0.5	* 0.037
hsCRP (mg/L)	7.7 (6–9.6)	4.2 (4–5.6)	* 0.002
Interleukin 6 (pg/mL)	41 ± 8	42 ± 9	0.47
CK18F (U/L)	247 ± 30	248 ± 37	0.8
TMAO (μmol/L)	3.9 ± 2.4	4.2 ± 1.9	0.42
Stool SCFA (mmol/L)	176 ± 36	174 ± 37	0.24
Fecal Calprotectin (μg/g)	140 ± 90	120 ± 80	0.3
Baseline CAP (dB/m)	295 ± 19	288 ± 21	0.1
Baseline LSM (kPA)	5 ± 2.5	4.4 ± 2.5	0.18
Baseline HSI	37.5 ± 2.6	36.5 ± 2.6	0.137
Baseline FLI	74.5 ± 4.5	75 ± 4.0	0.62
Baseline FIB-4	1.51 (1.05–1.76)	1.49 (1.1–1.87)	0.93
Baseline NFS	−1.17 (−1.97 to −0.4)	−1.11 (−2.01 to −0.37)	0.24

“*”—statistical significance reached, BMI—body mass index; ALT—alanine aminotransferase; AST—aspartate aminotransferase; GGT—gamma-glutamyl transferase; HbA1c—hemoglobin A1C; hsCRP—high-sensitivity C-reactive protein; CK18F—cytokeratin 18 fragment; TMAO—trimethylamine N-oxide; SCFA—short-chain fatty acid; CAP—Controlled Attenuation Parameter; LSM—liver stiffnes measurement; HSI—hepatic steatosis index; FLI—fatty liver index; FIB-4—Fibrosis 4; NFS—Non-Alcoholic Fatty Liver Disease Fibrosis Score.

## Data Availability

Study data available at 10.6084/m9.figshare.28509464.

## Abbreviations

MASLD—metabolic syndrome-related steatotic liver disease; LPS—lipopolysaccharide; SCFA—short-chain fatty acid; BMI—body mass index; ALT—alanine aminotransferase; AST—aspartate aminotransferase; GGT—gamma-glutamyl transferase; HDL-C—high-density lipoprotein cholesterol; HbA1c—*hemoglobin A1C*; hsCRP—high-sensitivity C-reactive protein; CKF18—cytokeratin 18 fragment; TMAO—trimethylamine N-oxide; SCFA—short-chain fatty acid; HSI—hepatic steatosis index; FLI—fatty liver index; FIB-4—Fibrosis 4; NFS—Non-Alcoholic Fatty Liver Disease Fibrosis Score; AIC—Akaike Information Criterion; BIC—Bayesian Information Criterion; SREBP—sterol regulatory element-binding protein; AMPK—adenosine monophosphate-activated protein kinase; GLP-1—glucagon-like peptide-1; CAP—Controlled Attenuation Parameter; LSM—liver stiffness measurement; FDR—false detection rate.

## Appendix A

**Table A1:** Dynamic of measured parameters.

Parameter	Sodium Butyrate	Calcium Butyrate	*p*
N	121	56	-
Δ ALT (U/L)	−0.3 ± 9.7	−0.2 ± 9.4	0.95
Δ AST (U/L)	0.6 ± 8.6	0.8 ± 7.1	0.88
Δ GGT (U/L)	−0.3 ± 6.3	0.6 ± 6.4	0.34
Δ hsCRP (mg/L)	0.5 ± 2.1	0.8 ± 2.4	0.36
Δ Interleukin 6 (pg/mL)	1.5 ± 9.6	0.8 ± 10.8	0.68
Δ CK18F (U/L)	1.3 ± 22.4	−5.5 ± 22.8	0.065
Δ TMAO (μmol/L)	10 ± 6	0.1 ± 1.3	0.001
Δ Fecal Calprotectin (μg/g)	3 ± 26.6	9 ± 31.9	0.16
Δ CAP (dB/m)	2 ± 11.8	0.2 ± 18.1	0.42
Δ LSM (kPA)	−0.09 ± 0.6	0.2 ± 0.6	0.22
Δ HSI	0 ± 1.33	0.1 ± 1.2	0.61
Δ FLI	−4.1 ± 12.3	0.5 ± 15.8	0.036
Δ FIB−4	0.02 ± 0.06	0.023 ± 0.04	0.81
ΔNFS	0.015 ± 0.03	0.016 ± 0.04	0.88

**Table A2:** Linear mixed-effects model summary: fixed effects for time × treatment interaction.

Outcome Variable	Estimate	Std. Error	95% CI	*p*-Value
CAP (dB/m)	−0.95	1.21	[−3.30, 1.40]	0.680
LSM (kPa)	−0.20	0.34	[−0.90, 0.50]	0.550
TMAO (μmol/L)	−1.32	0.52	[−2.35, −0.29]	* 0.021
Fecal Calprotectin (μg/g)	−18.90	8.60	[−35.70, −2.10]	* 0.031
HIS	−0.05	0.11	[−0.27, 0.17]	0.610
FLI	−2.35	1.16	[−4.62, −0.08]	* 0.047
FIB-4	−0.004	0.033	[−0.068, 0.06]	0.9
NFS	0.015	0.067	[−0.117, 0.148]	0.82

“*” statistical significance reached.

**Table A3:** Regression of microbiota vs. endpoint Controlled Attenuation Parameter.

Family/Genera	β-Coefficient	Raw *p*	FDR-Adjusted *p*
Enterococcaceae	−37.023	0.01	0.278
Veillonellaceae	300.079	0.104	0.739
Coriobacteriaceae	199.65	0.109	0.739
Enterobacteriaceae	224.791	0.245	0.742
Prevotellaceae	136.228	0.302	0.742
Bacteroidaceae	151.436	0.42	0.81
Streptococcaceae	17.595	0.636	0.959
Lachnospiraceae	6.783	0.639	0.959
Bifidobacteriaceae	11.13	0.824	0.99
Ruminococcaceae	−0.737	0.97	0.99
Lactobacillaceae	−0.582	0.974	0.99
Enterococcus	−31.042	0.063	0.739
Blautia	31.603	0.243	0.742
Ruminicoccus	53.649	0.287	0.742
Catenibacterium	−49.063	0.29	0.742
Subdoligranum	35.249	0.511	0.862
Streptococcus	14.144	0.706	0.99
Holdemanella	8.714	0.856	0.99
Ruminicoccus T	4.436	0.963	0.99
Faecalibacterium	2.45	0.966	0.99
Lactobacillus	−0.582	0.974	0.99

## Appendix B

### Stool Microbiome Determination Details

Patients were provided with stool containers and instructed to bring fresh stool samples (collected within 6 h) to the center. These samples were stored at −80 °C prior to microbiome profile analysis. Total genomic DNA was extracted from the frozen fecal samples using the ZR Fecal DNA MiniPrep Kit (Zymo Research Corp., Irvine, CA, USA), following the manufacturer’s instructions. The isolated DNA from all samples was stored at −20 °C after measuring DNA concentration using PicoGreen on a Qubit fluorometer (ThermoFisher/Invitrogen, Waltham, MA, USA). All samples were diluted to a concentration of 5 ng/μL in a final volume of 10 μL and used for 16S rRNA amplicon sequencing, targeting the V3–V4 hypervariable region. Paired-end sequencing was conducted on the NovaSeq 6000 PE250 Illumina platform by Novogene Co., Ltd. (Hong Kong, China). The libraries were subsequently sequenced on an Illumina MiSeq platform (Illumina, San Diego, CA, USA) following standard protocols established by Majorbio Bio-Pharm Technology Co., Ltd. (Shanghai, China). The raw reads were demultiplexed and quality-filtered using Usadellab Trimmomatic (Aachen, Germany), and analyzed with the Quantitative Insights Into Microbial Ecology (QIIME) pipeline as described by Caporaso et al. [52]. Operational taxonomic units (OTU) clustering and species annotation were performed, with sequence analysis conducted using Uparse software (Uparse v7.0.1001, Tiburon, CA, USA) [53]. Sequences with 97% or greater similarity were grouped into the same OTUs. For each representative sequence, the mothur software (Version 1.35.1) was utilized against the small subunit ribosomal RNA database of the SILVA Database for species annotation across various taxonomic levels (kingdom, phylum, class, order, family, genus, species), using a threshold of approximately 0.8 to 1. OTU abundance information was normalized based on the sequence number corresponding to the sample with the fewest sequences [54,55]. The Shannon and Simpson indexes were calculated in R to measure alpha diversity. Beta diversity was assessed using weighted UniFrac as a distance measurement, with the “anosim” p-method applied, following 999 permutations to determine differences between groups. Differences in β-diversity were visualized using principal coordinates analysis biplots.
